# Exploring differences between groove and catchiness

**DOI:** 10.3389/fpsyg.2025.1642561

**Published:** 2025-10-01

**Authors:** Toni A. Bechtold, Ben Curry, Maria A. G. Witek

**Affiliations:** ^1^Deparment of Music, University of Birmingham, Birmingham, United Kingdom; ^2^Lucerne School of Music, Lucerne University of Applied Sciences and Arts, Lucerne, Switzerland

**Keywords:** groove, catchiness, urge to move, music psychology, AI music

## Abstract

**Introduction:**

Groove and catchiness play a significant role in popular music, and a series of studies has shown that they are positively related. In this study, we explored the limits of this relationship: when are groove and catchiness not related, and which musical factors promote one but not the other? To address the first question, we focused on duration: groove (an urge to move along to music) is thought to require representation of meter and repetition, and thus a certain duration, while catchiness is thought to act within fractions of a second.

**Methods:**

In a listening experiment, 92 participants rated 54 AI-generated music excerpts that varied in style, tempo, and duration (1 second and 10 seconds) on urge to move, pleasure, and catchiness. Additionally, they assigned the stimuli to one or more of 13 popular music styles and completed a recognition task. To examine the influence of musical characteristics, we measured 18 audio features of the music. We analyzed these data using *t*-tests, correlation analyses, and Bayesian regression models to assess the relationships between listener responses, stimulus conditions, and musical features.

**Results and discussion:**

Even the 1-second excerpts elicited some urge to move—though less than for 10-second excerpts, while catchiness ratings were on average similar across durations. Catchiness and urge to move ratings were correlated even in the 1-second condition. These findings suggest a complex, reciprocal relationship between catchiness and the urge to move in listeners, which we partly explain through a distinction between ‘transient’ and ‘sustained’ catchiness. We identified some music-related factors that affected only one of the two ratings: rhythmic information and tempo affected urge to move only. In contrast, recognizability substantially increased catchiness but had little effect on the urge to move. Four out of 13 popular music styles (as perceived by participants) affected catchiness but not the urge to move, while three out of 18 audio features affected one but not the other. In summary, while we found further support for a positive relationship between groove and catchiness, this relationship is constrained by duration and certain musical characteristics, which can affect the two responses to music differently.

## Introduction

Music can affect us in many ways: it can improve our mood, make us move, stick in our minds, and make us sing along. Previous research has shown that, in popular music, two such reactions tend to go hand in hand and benefit each other: groove and catchiness (Bechtold et al., 2023, 2024, 2025a). From the perspective of popular music creators, groove and catchiness are fundamental to popular music: they often constitute goals of this music and are central to its appeal and success. Understanding the relationship between groove and catchiness can deepen our insight into how music shapes affective and sensorimotor experiences. It can also inform how we conceptualize music-induced pleasure, movement, and memory, and help explain how these intertwined responses influence our everyday interactions with music. However, not all popular music is equally catchy and groovy—Céline Dion’s “My heart will go on” is certainly memorable but not particularly movement-inducing, whereas Fela Kuti’s “Teacher Do not Teach Me Nonsense” exemplifies groove without melodic catchiness. Where does the relationship between these two phenomena end and how distinct are their underlying psychological constructs? This study investigates these questions by exploring differences between groove and catchiness, focusing on the time and rhythmic information that each requires to unfold, as well as the musical factors that may promote one but not the other.

### Groove

There are multiple definitions of groove in music psychology. Some studies described a complex multidimensional experience (Pfleiderer, 2010; Bechtold et al., 2023; Duman et al., 2024) with important individual differences (Hosken, 2020), but in most studies, groove has been understood and/or measured as pleasurable urge to move to music (Janata et al., 2012), or simply the urge to move to music (Madison, 2006; Senn et al., 2023a, 2023b, 2024; Bechtold et al., in press). Conceptually, this means that groove is understood as felt in the body (Roholt, 2014; Witek, 2017; Bechtold et al., 2023) – it is a process in the listener, not in the music.

Many studies have investigated what aspects of music foster an urge to move, with a sense of meter often implied as necessary. For example, Witek (2017) described groove as creating a multi-sensory representation of the musical meter in the body, and rhythmic complexity and syncopation (i.e., a disruption of a meter) have been shown to affect groove (Witek et al., 2014, 2017, 2020; Matthews et al., 2019; Spiech et al., 2022; Stupacher et al., 2022a; Sioros et al., 2022; Duncan and Orgs, 2024): music that occupies a sweet spot between boring and overly complex likely provokes movement (although this has been somewhat challenged by Senn et al., 2024). Some studies have explained the effect of syncopation (and the urge to move in general) with the theory of predictive coding of rhythmic incongruity (Vuust et al., 2018; Stupacher et al., 2022b; Senn, 2023; Senn et al., 2024). This theory holds that human brains try to minimize prediction errors, and thus constantly update their inner model of the music in response to auditory input. As perceived metric structure influences when and how strongly listeners expect events (Vuust and Witek, 2014) and thus the inner model, this approach also relies on the listener’s extraction of pulse, meter, or regularity. In sum, a sense of meter seems important for groove, implying that the music needs to have a certain duration and regularity.

Humans can detect violations of a meter almost instantly (Schultz et al., 2013; Bouwer et al., 2020), but no studies to date have examined how quickly humans can infer the beat or pulse from music—which likely depends on the musical characteristics and the listener – although it has been assumed to be rapid (Grahn and Rowe, 2013). For example, one measure may suffice under beneficial circumstances (Merchant et al., 2015). Likewise, a temporal threshold for groove has not yet been examined. This is important because, if syncopation is a factor for groove, then experiencing groove requires first finding the beat, followed by enforcing and disrupting it. Musically speaking, this means it requires at least several beats or even bars. This aligns with musicological research that has emphasized the importance of repetition for groove (Feld, 1988; Zbikowski, 2004; Danielsen, 2006) or empirical research that has investigated the role of patterns, which also require several beats to unfold (Senn et al., 2018; Sioros et al., 2022).

Audio features have also been studied for their influence on the urge to move. For example, the clarity of the pulse or beat, the music’s percussiveness, perceived loudness, brightness, as well as spectral flux (particularly in the bass frequencies) have shown positive effects (Burger et al., 2013; Stupacher et al., 2016; Spiech et al., 2022; Düvel et al., 2022; Bechtold et al., 2025b; Bechtold et al., in press; Duncan and Orgs, 2024). Studies that investigated the effect of tempo on the urge to move found either an optimal tempo range between 100 and 120 BPM (Etani et al., 2018) or that faster tempos are preferred (Jerjen et al., 2024). A few studies looked at the influence of broad musical style families (Senn et al., 2021; Stupacher et al., 2023) and found that funk elicits a stronger urge to move than pop and rock.

Aside from music-related factors, studies have shown how individual differences (familiarity, musical expertise/training, music and dance preferences) influence whether people are propelled to move to music (Janata et al., 2012; Senn et al., 2018, 2021; Matthews et al., 2019, 2022; Kowalewski et al., 2020; Bechtold et al., 2024). Two series of studies have examined the urge to move in relation to other perceptual or cognitive processes: one revolves around a psychological model of groove, and has investigated how energetic arousal, listening pleasure, rhythmic interest, and the representation of temporal regularity influence the urge to move (Senn et al., 2019, 2023a, 2023b, 2024; Bechtold et al., in press; Jerjen et al., 2024). The other, which includes the present study, has looked at its relation with catchiness (Bechtold et al., 2023, 2024, 2025a).

### Catchiness

Compared to groove, the concept of catchiness is less well defined and researched. In many studies, the term was used synonymously with recognizability, memorability, or sticking in mind (Russell, 1987; Honing, 2010; Van Balen, 2016; Grevler, 2019), or defined as long-term musical salience (Burgoyne et al., 2013). Conceptually, this means that catchiness is thought to be a property of the music, and ultimately traceable to the sound itself. It has been found that catchiness plays a role in involuntary musical imagery (INMI; for an overview see Liikkanen and Jakubowski, 2020) and hooks (Burns, 1987; Byron and O’Regan, 2022). While INMI research has acknowledged the importance of individual differences, such as familiarity, music preferences, gender, personality, and musical training (Floridou et al., 2015; Beaman and Williams, 2013; Beaty et al., 2013; Hyman et al., 2013; Campbell and Margulis, 2015), it has also treated catchiness as a musical property (Moeck et al., 2018). In contrast, Bechtold et al. (2023, p. 353) found that catchiness is a perceptual phenomenon, which is also the understanding used in this study: “a multi-dimensional quality that depends on the listener’s perception and experience of music, in which memorization and positive affect are central, and engagement, immediacy, and clarity are other aspects.” Conceptually, this means that catchiness forms in perception and is experienced; it is a cognitive process that, while triggered by the music, happens in the listener, not in the music.

Given the view of catchiness as a musical property, research on catchiness, memorability, and hooks has often focused on musical aspects, specifically melody (Kronengold, 2005; Pawley and Müllensiefen, 2012; Kramarz, 2014; Jakubowski et al., 2017; Grevler, 2019; Silas and Müllensiefen, 2023). Some studies have emphasized that other musical parameters, including rhythm and non-textual aspects (such as timbre or timing), can enhance catchiness (Burns, 1987; Honing, 2010; Byron and O’Regan, 2022). For perceived catchiness, individual differences are important by definition: Bechtold et al. (2024) found that music preferences, dance preferences, familiarity, and expertise play a role, corroborating qualitative results from Bechtold et al. (2023).

### The relationship between groove and catchiness

A potential relationship between groove and catchiness has been hinted at, for example with observations that INMI is often accompanied by movement (Campbell and Margulis, 2015; Floridou et al., 2015; Jakubowski et al., 2015). While this relationship had not been directly examined before the current series of studies, doing so allows us to clarify whether and how groove and catchiness emerge together, which is essential for understanding how music becomes engaging, memorable, or generally impactful. A qualitative study explored the relationship as understood by music creators (Bechtold et al., 2023), and two quantitative studies tested it in individual patterns (Bechtold et al., 2024) and in polyphonic music (Bechtold et al., 2025a). All three demonstrated a positive relationship between groove/urge to move and catchiness. The qualitative results suggested that groove and catchiness interact positively, and in some cases can even fuse into an indivisible experience. Bechtold et al. (2024) found empirical support for a causal link: catchiness increases listening pleasure, which in turn increases the urge to move. Bechtold et al. (2025a) found that they reinforce one another when patterns are combined, which makes them hard to disentangle in polyphonic music. In Bechtold et al. (2024), listener-related factors (preferences, familiarity, expertise) were identified as mutually promoting factors for catchiness and the urge to move, while musical factors (whether a pattern is a drum beat, bass line, or keyboard riff) showed diverging effects. Successful recognition of the music was associated with increased urge to move and catchiness alike. In summary, all three studies evidenced similarities and positive relationships between groove and catchiness, and only a few concrete differences – all based on music structure.

### Potential differences and hypotheses

Where does the relationship end? Groove and catchiness are not the same phenomenon. Understanding the limits of the relationship is crucial for distinguishing their underlying psychological and musical mechanisms, and for explaining why some music evokes one response but not the other. Our previous qualitative results led to a theory of independence when one of them is not or hardly present. This can be seen in our previous quantitative studies (Bechtold et al., 2024, 2025a), which found correlations suggesting a general relationship, though their limited strength indicates that the two can also vary independently. So where exactly are the conceptual differences, and is it possible to identify musical characteristics that promote one but not the other?

In this study, we explore these two questions in six steps. For conceptual differences (H_I_), we focus on the temporal domain. As detailed above, groove relies on a representation of meter or regular rhythmic structure, which suggests it requires a certain duration, otherwise these cannot be inferred. The exact threshold is unknown, but we expect a minimum of two bars or 3–8 s in common tempos. In contrast, catchiness has been described as immediate (Bechtold et al., 2023). Music can be recognized in fractions of a second (Krumhansl, 2010) and recognition speed has been used as a measure of catchiness (Burgoyne et al., 2013). Thus, we expect to see a difference between groove and catchiness when the musical stimuli are of short duration (1 s) and contain a minuscule amount of rhythmic information (≤ 2 beats): such music—below our estimated threshold – should not elicit a noteworthy urge to move, if at all, while catchiness should be similar across durations (H_Ia_). We expect that even a slight increase of the rhythmic information (1.5 beats vs. 2 beats) increases the urge to move, while not affecting catchiness (H_Ib_).

Past studies (Bechtold et al., 2023, 2024) have shown that musical properties can lead to different outcomes for urge to move and catchiness (H_II_).

- Musical tempo has been identified as a factor for urge to move (Etani et al., 2018; Jerjen et al., 2024; Bechtold et al., 2025b), but its effect on catchiness has not been examined. We expect faster stimuli (120 BPM) to elicit higher urge to move than slower stimuli (90 BPM), while catchiness remains unaffected (H_IIa_).
- Musical styles are often loosely defined, but they vary in a range of characteristics and parameters. Studies on groove have shown a stronger association with funk styles than with rock styles (Senn et al., 2021; Stupacher et al., 2023), and several groove studies focused on Electronic Dance Music (EDM, Wesolowski and Hofmann, 2016; Lustig and Tan, 2019; Duncan and Orgs, 2024). There is no equivalent result for catchiness, but some styles (e.g., pop) seem likely to be more closely associated with catchiness than others based on theoretical considerations (Rösing, 1996; Kramarz, 2014). We expect that style affects urge to move and/or catchiness, but in different strengths: funk and EDM show increased groove, pop shows increased catchiness (H_IIb_).
- The recognizability of music is integral to catchiness (see definitions), but rarely associated with the urge to move. Hence, we expect a larger positive effect of recognizability on catchiness than on the urge to move (H_IIc_).
- We compare whether specific audio features affect either urge to move, catchiness, or both (H_IId_). For example, increased spectral flux has been associated with higher urge to move (Stupacher et al., 2016; Bechtold et al., 2025b; Bechtold et al., in press), while it has been found to hinder recognition (Kuiper et al., 2021). In general, we expect some differences in associations: since research on groove has focused on rhythm, rhythmic variables (e.g., pulse clarity, percussiveness) are expected to influence the urge to move. In contrast, as catchiness has often been linked to pitch-related features, we expect melodic variables (e.g., key strength, tonality) to influence catchiness.

## Materials and methods

### Participants

We recruited 92 participants (43 female, 48 male) on the Prolific platform (www.prolific.com), aged between 18 and 58 (mean = 28.217 years, SD = 8.390), and living in Europe (57), South Africa (25), USA (7), Mexico, Chile, and Kenya (1 each). They assessed their musical expertise rather low (mean = 24.804, SD = 30.800) on a scale from 0 (music listener) to 100 (professional musician). Half of the participants never played an instrument (47), the others were mostly pianists (13), guitarists (12), or singers (8). Participants showed some affinity for popular music styles (on a scale from 0 to 6: mean = 4.074, SD = 0.705) and for dancing (on a scale from 0 to 6: mean = 4.277, SD = 1.438).

### Stimuli

We aimed for short music excerpts that represent popular music from a variety of styles but were unfamiliar to the participants. Very short excerpts of familiar music would undermine the design of the study, as recognition would prompt participants to mentally continue the song and base their ratings on more than just the excerpt itself. Hence, we used music created by the Suno AI (version 3.5). Suno creates short songs (usually between 1 and 4 min) based on concise prompts about genre or style, instrumentation, musical gesture, or lyrics. The songs vary in quality and adherence to the prompts, as some styles can be reproduced better than others, but often resemble full-band music with sections that sound ecologically valid.

We wanted three songs each from nine different musical styles in two different tempos, leading to a total of 54 songs. Our prompts focused on the desired styles (or substyles), and sometimes basic specifications (e.g., ‘fast’, ‘slow’, ‘ballad’, or ‘80s’). We discarded tracks featuring vocals to avoid any influence of lyrics. For some styles, it was easy to get music in the targeted tempos, while others (e.g., fast HipHop/Contemporary R&B or slow Alternative/Punk) required many attempts. In the end, we created 250 songs and selected 54 based on perceived overall quality and proximity to the target tempos. In some cases, the musical style differed from the prompt (e.g., “acoustic pop for a party” created a country song), and we reassigned these to an appropriate style. Our main goal regarding styles was to include a varied and broad set of music with many different characteristics – and not to have the best possible representation of a style, as we use a different participant-assigned style variable in the analysis.

We proceeded to select a 10-s excerpt of each song for the long stimuli condition. We aimed for 120 and 90 BPM as tempos, as these represent 1.5 and 2 beats in the short duration condition. We identified the beat and thus the tempo of each stimulus based on the backbeat structure. We normalized the stimuli for loudness and changed their tempo to either 120 or 90 BPM in Audacity (version 2.4.2). Then, we extracted the first second for the short stimuli condition. We took special care that the first second was representative of the following 9 s, i.e., we avoided new song sections, large differences in instrumentation, and surprising elements, such as breaks or fills. The resulting 108 audio stimuli (3 × 9 styles x 2 tempos x 2 duration conditions) and respective prompts can be found in the online repository.^1^

### Measures

#### Ratings of experiences: urge to move, pleasure, and perceived catchiness

We measured urge to move and pleasure with the Experience of Groove Questionnaire (EGQ, Senn et al., 2020). We used its latest version (Senn et al., 2024), in which participants rate their experienced urge to move (3 items) and listening pleasure (4 items) on 7-point Likert scales. We measured perceived catchiness with the questionnaire used in Bechtold et al. (2024) and Bechtold et al. (2025a) that features 4 items and the same scales. We slightly changed all items and replaced ‘this music’ with ‘this music clip’. This seemed necessary for the 1-s stimuli to ensure that participants rated their experience in response to what they actually heard, rather than similar music, a general impression, or an imagined continuation, which was also emphasized in the instructions. The respective items of each scale were averaged to create the condensed ratings urge to move_10s_, urge to move_1s_, pleasure_10s_, pleasure_1s_, catchiness_10s_, and catchiness_1s_, which are used for H_I_, while only the 10-s ratings are used for H_II_. Pleasure has been shown to be integral for both groove and catchiness, even serving as a link between them (Bechtold et al., 2023, 2024), making it less suitable for exploring differences between them. Hence, we only report analyses for pleasure when relevant for their relationship, and otherwise focus on urge to move and perceived catchiness.

#### Tempo

We created stimuli in two tempos: 90 and 120 BPM. We used this measure for H_IIa_, examining whether tempo affects the 10-s stimuli. If we assume that 1 s is too short for inferring meter, the tempo of the music cannot be determined by the listener for music of this duration. Therefore, we use a different interpretation for H_Ib_: for the 1-s stimuli, 90 and 120 BPM correspond to 1.5 and 2 beats of music. Hence, we use tempo manipulation to create conditions with different amounts of rhythmic information.

#### Style assignments and style bias

We asked participants to optionally assign each 10-s stimulus to one or more of 13 popular music styles. When they assigned multiple styles, we weighted the assignments: if a participant chose only Pop/Mainstream, it was assigned a value of 1, but if they chose both Pop/Mainstream and EDM/Dance, it counted as 0.5 each. We also calculated a style bias for each observation, which is a participant’s disposition towards the style they assigned the music to (Senn et al., 2018), or the average disposition across selected styles if they selected multiple styles. Both measures inform H_IIb_.

#### Recognizability

We measured recognizability in the second part of the experiment (see below), when participants had already heard the 1-s stimuli and were rating the 10-s stimuli. We asked them whether they thought they had heard an excerpt of the current 10-s stimulus before, on a scale from 0 (definitely not) to 6 (definitely). This measure is analyzed for H_IIc_.

#### Audio features

We investigated which audio features promote or hinder urge to move and catchiness (H_IId_). Several musical measures have previously been used for predicting urge to move or catchiness/memorability. For the latter, studies based their analyses on MIDI or symbolic representation of monophonic music (Van Balen, 2016; van Nieuwenhuijsen et al., 2020; Silas and Müllensiefen, 2023). This kind of data is not available for our stimuli, and analyses would differ from previous studies due to the full-band stimuli. For urge to move, several studies have examined features measured directly from the audio (Burger et al., 2013; Stupacher et al., 2016; Spiech et al., 2022; Düvel et al., 2022; Bechtold et al., 2025a,b). We opted for this method and measured 18 audio features of the 10-s stimuli with the MIR Toolbox (version 1.8.1) in MATLAB (version R2023b). We selected variables that have previously been associated with the urge to move (e.g., pulse clarity and sub-band flux no. 2), and added variables that we thought likely to influence catchiness, as they resemble measures from studies using symbolic input (e.g., key strength, tonality). The commands and a short description of each measure can be found in the online repository.

### Procedure

We used the SoSci Survey platform^2^ for the experiment. First, the participants were informed about the study and received instructions, then they gave informed consent by clicking a button. On the following page, participants answered questions on their personal background. Then, after a musical example to adjust the volume to a comfortable level, the main part of the experiment started. A pseudo-randomization assigned each participant 18 stimuli (9 styles x 2 tempos). In the first part, they heard the 1-s versions and rated one per page on catchiness. Afterwards, they rated the same stimuli on urge to move and pleasure, with both ratings presented together on the same page. In the second part of the experiment, they heard the 10-s versions. The stimuli were again presented one per page, first with the catchiness questionnaire and the recognizability item, then again with the EGQ and style assignments. We recruited participants until each stimulus was rated 30 times. As each participant rated only a third of the stimuli, this meant we needed at least 90 participants. The study was approved by the University of Birmingham’s Humanities and Social Sciences Ethical Review Committee (ERN_20–0007) and adhered to the Declaration of Helsinki. The experiment took 30 min on average (SD = 8.854). Participants were remunerated with £6.

### Statistical analysis

We performed all statistical analyses in R (version 4.4.2) and RStudio (version 2024.09.1). We used a Bayesian framework throughout, which quantifies evidence in favor or against a hypothesis with a Bayes Factor (BF). Table 1 gives the interpretations of BFs based on Lee and Wagenmakers (2014), and how these are treated and represented in the present study. We report BFs up to 1,000, with larger values shown as > 1,000. Decimal places are included only for values below 100.

**Table 1:** Interpretation scheme for Bayes factors (Lee and Wagenmakers, 2014) with the interpretation used in the present study and the associated visual representation.

Bayes factor	Evidence category	In this study
>100	Extreme evidence for H_1_	**Confirmed effect (bold)**
30–100	Very strong evidence for H_1_	**Confirmed effect (bold)**
10–30	Strong evidence for H_1_	*Trend (italics)*
3–10	Moderate evidence for H_1_	No effect (plain)
1–3	Anecdotal evidence for H_1_	No effect (plain)
1	No evidence	No effect (plain)
1/3–1	Anecdotal evidence for H_0_	No effect (plain)
1/10–1/30	Moderate evidence for H_0_	No effect (plain)
1/30–1/10	Strong evidence for H_0_	*Trend (italics)*
1/100–1/30	Very strong evidence for H_0_	**Confirmed effect (bold)**
<1/100	Extreme evidence for H_0_	**Confirmed effect (bold)**

For H_Ia_ and H_Ib_, we compared the means of ratings with Bayesian t-tests from the BayesFactor package (Morey et al., 2022) to uncover potential differences between the 1-s and 10-s stimulus conditions, and the 1.5 beat and 2 beat rhythmic information conditions. We calculated correlations between the ratings with the correlations package (Makowski et al., 2020) for H_Ia_, which additionally shows the relationship between urge to move and catchiness in the duration conditions. We compared the strength of two correlations with the BFpack package (Mulder et al., 2021).

We calculated Bayesian regression models to investigate all hypotheses and followed the same procedure throughout. First, we created the respective models with the brms package (Bürkner, 2017), using flat priors for data driven models (except where noted otherwise), while specifying different random term structures: baseline model (BL), by-participant intercept (PI), by-stimulus intercept (SI), both intercepts (PI/SI), by-participants slope (PS), by-stimulus slope (SS), by-participant slope and by-stimulus intercept (PS/SI), both slopes (PS/SS). Then, we compared the models based on their expected log pointwise predictive density (ELPD) differences with the loo package (Vehtari et al., 2023). The ELPD weights prediction accuracy against increased model complexity, suggesting a model that is most efficient. We regard ELPD differences < 4 as negligible and chose the less complex model in these cases, otherwise we went for the model with the highest ELPD. We calculated the coefficient of determination with the performance package (Lüdecke et al., 2021) and report the marginal R^2^, i.e., the value for the fixed effects of the model (R^2^_m_). To quantify the evidence for a variable’s effect, we employed directional hypothesis tests from the brms package (*B* > 0). We inverted the hypothesis for negative effects (*B* < 0), so that all BFs > 1 are evidence towards having an effect. To compare effect sizes across conditions (1 s vs. 10s) or rating types (urge to move vs. catchiness), we calculated a single regression model using a long-format structure where both outcome values were combined into one column. The model included the predictor of interest, a categorical variable indicating the condition or rating type, and their interaction. This allowed direct estimation of effect size differences via the interaction term, while accounting for shared variance across outcomes.

## Results

### Conceptual differences

#### Stimulus duration

T-tests provided no evidence for differences between the mean ratings for the 10-s and 1-s conditions for catchiness and pleasure (Table 2). For urge to move, the 1-s condition was on average rated lower than the 10-s condition.

**Table 2:** Means for the 3 rating scales in the 10s and 1 s conditions, and Bayes factors for the *t*-test whether the two means for the same rating scale are different.

DV	10s	1 s	BF (*t*-test)
Catchiness	3.585	3.460	0.738
Urge to move	3.177	2.856	**> 1,000**
Pleasure	3.535	3.398	0.829

Bayes factors > 10 are shown in italics, and > 30 in bold.

**Figure 1:**
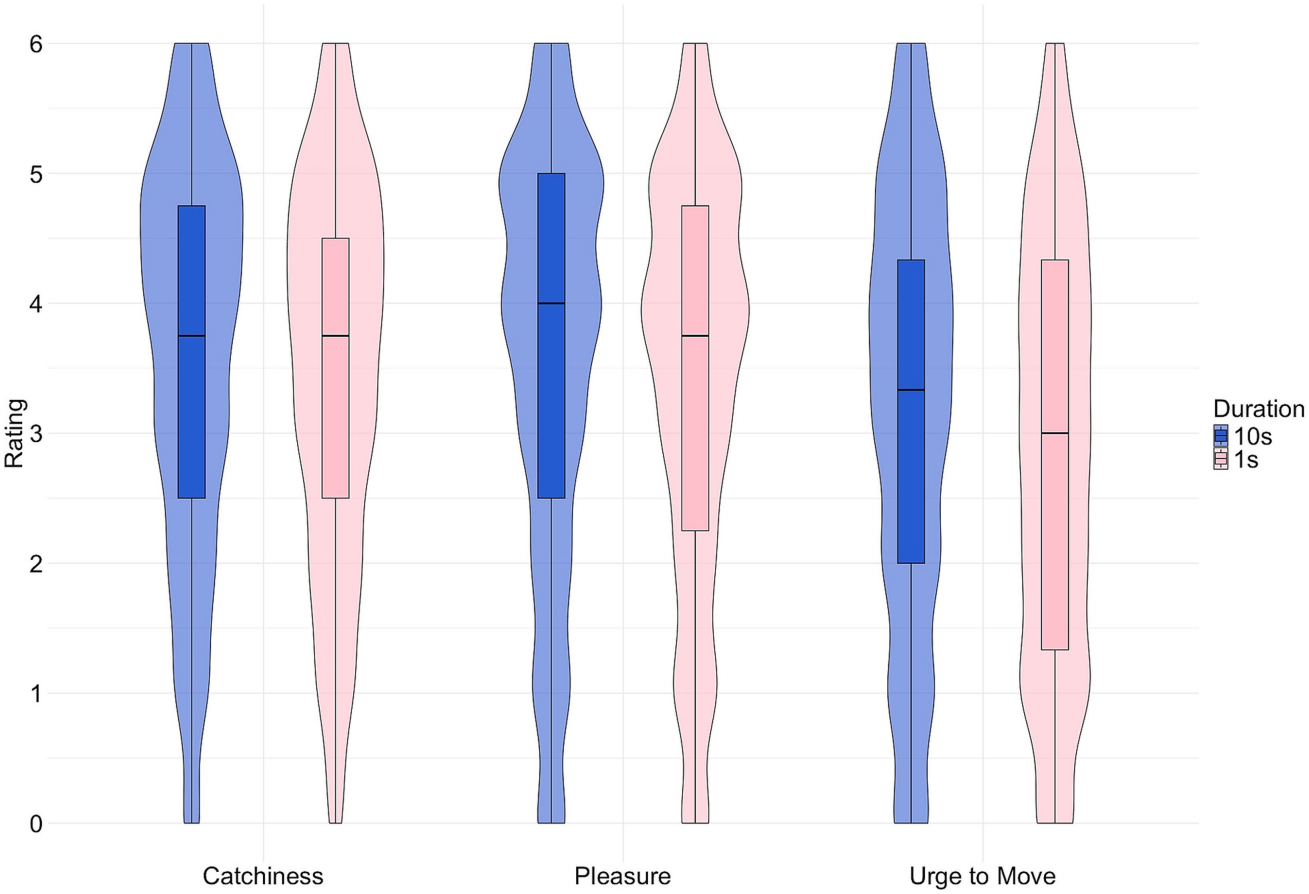
Boxplot and density of the catchiness (left), pleasure (middle), and urge to move (right) ratings, with duration in blue (10s) and pink (1 s).

Figure 1 shows the density and quartiles of ratings for catchiness, pleasure, and urge to move in the two duration conditions. We can observe great similarity between the two catchiness ratings, and between the two pleasure ratings (albeit the mean for pleasure_1s_ is nominally lower), but urge to move_1s_ is much flatter than urge to move_10s_ and all others. Taken together, means and distributions suggest a negative influence of short stimulus duration on urge to move but not on pleasure or catchiness. However, the ratings for urge to move_1s_ show some variation and are not exclusively low.

The correlations between the respective ratings in the two duration conditions revealed additional implications. There is a strong positive correlation for urge to move (Table 3), which is stronger than the moderate positive correlation for catchiness (BF > 1,000). This relatively weak correlation for catchiness indicates that, while neither 1-s nor 10-s ratings are generally higher than each other, ratings are not stable across conditions. We found positive correlations between urge to move and catchiness in both duration conditions. As expected, that correlation is stronger for the 10-s condition compared to the 1-s condition (BF > 1,000). For pleasure, there is also a strong positive correlation.

**Table 3:** Correlations between ratings in different conditions.

Ratings	*ρ*	95% CrI	BF
Catchiness_10s_ ~ Catchiness_1s_	0.432	0.392, 0.471	**> 1,000**
Move_10s_ ~ Move_1s_	0.548	0.513, 0.581	**> 1,000**
Pleasure_10s_ ~ Pleasure_1s_	0.564	0.532, 0.595	**> 1,000**
Catchiness_10s_ ~ Move_10s_	0.487	0.452, 0.526	**> 1,000**
Catchiness_1s_ ~ Move_1s_	0.402	0.363, 0.441	**> 1,000**

Bayes factors > 10 are shown in italics, and > 30 in bold.

Next, we calculated regression models to test implied causalities (Table 4). The models show that we can generally predict the 10-s ratings from the 1-s ratings, and a model fit for comparing effect sizes showed that predictions are more accurate for urge to move than for catchiness (BF = 14.717). Furthermore, we can predict urge to move from catchiness. Predictions across durations and variables are also possible. For example, catchiness_1s_ can predict urge to move_10s_.

**Table 4:** Models for urge to move and catchiness in different conditions.

DV	IV	*B*	SE	95% CrI	BF	*R* ^2^_m_	Structure
Catchiness_10s_	Catchiness_1s_	**0.375**	0.034	0.307, 0.442	**> 1,000**	**0.142**	PS/SS
Urge to move_10s_	Urge to move_1s_	**0.432**	0.035	0.362, 0.500	**> 1,000**	**0.205**	PS/SS
Urge to move_1s_	Catchiness_1s_	**0.335**	0.032	0.271, 0.398	**> 1,000**	**0.096**	PS/SI
Catchiness_10s_	Urge to move_10s_	**0.396**	0.029	0.338, 0.453	**> 1,000**	**0.200**	PS/SS
Urge to move_10s_	Catchiness_1s_	**0.316**	0.026	0.265, 0.367	**> 1,000**	**0.081**	PI/SI

DV, dependent variable; IV, independent variable, PI, by-participant intercept; SI, by-stimulus intercept; PS, by-participant slope; SS, by-stimulus slope. Bayes factors reflect evidence for directional hypotheses B > 0.

Bayes factors > 10 are shown in italics, and > 30 in bold.

Contrary to the near-zero expected rating, urge to move_1s_ showed substantial values. Since this urge to move can be predicted to some extent by catchiness_1s_, we conducted a mediation analysis to test whether these values can be explained with a pathway identified in Bechtold et al. (2024) for longer excerpts: catchiness affects urge to move through pleasure. For the 1-s ratings, the mediation analysis (using a model with by-participant slope and by-stimulus intercept) showed that 79.7% of the effect of catchiness_1s_ on urge to move_1s_ is mediated by pleasure_1s_.

#### Amount of rhythmic information

**Figure 2:**
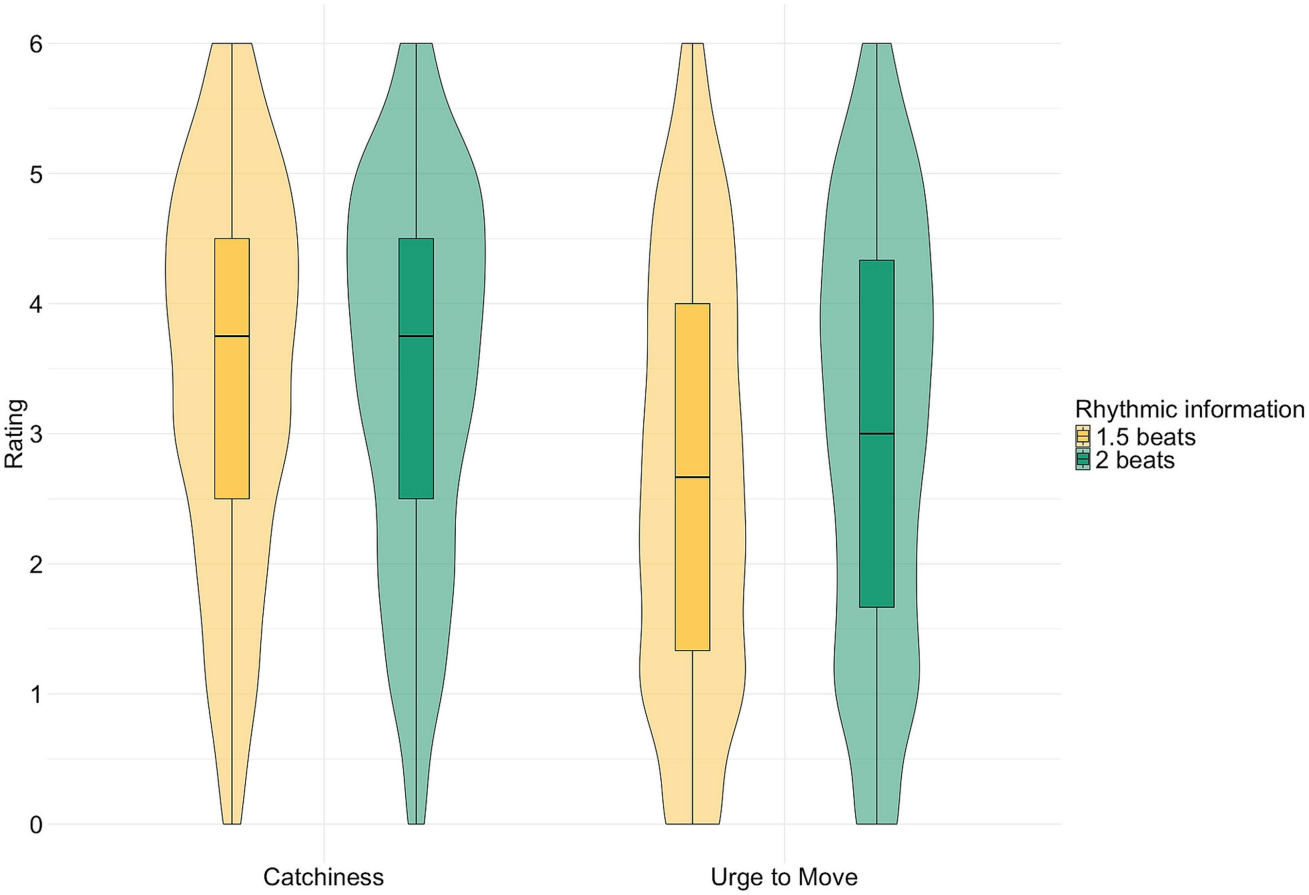
Boxplot and density of the catchiness (left) and urge to move (right) ratings, with rhythmic information in yellow (1.5 beats) and green (2 beats).

The 1-s stimuli have two different conditions based on the tempo manipulation. As tempo can hardly be inferred in such a short duration, we interpret the stimuli as encompassing 1.5 beats or 2 beats, i.e., differing in amount of rhythmic information. Figure 2 shows great similarity between catchiness ratings, while urge to move ratings differ both from the catchiness ratings and from each other. Table 5 gives the mean ratings per condition, which were similar for catchiness, but not for urge to move: in the 2 beats condition, urge to move ratings were higher than in the 1.5 beat condition. The mean rating for the 1-s clips encompassing 2 beats (i.e., 120 BPM) even approaches the mean of the 10-s clips at 120 BPM (3.314), but the longer clips were still rated higher than the shorter clips (BF_t-test_ = 17.779).

**Table 5:** Means for urge to move and catchiness with the 1 s stimuli grouped by 1.5 and 2 beat rhythmic information conditions, and Bayes factors for the *t-*test whether the two means for the same rating scale are different.

DV	1.5 beats	2 beats	BF (*t*-test)
Catchiness_1s_	3.462	3.458	*0.055*
Urge to move_1s_	2.674	3.038	**> 1,000**

Bayes factors > 10 are shown in italics, and > 30 in bold.

We followed this up with regression models that predict the 1-s ratings based on the rhythmic information condition (Table 6). This confirmed the results above: there was no effect on catchiness but urge to move benefited from having more rhythmic information.

**Table 6:** Models for urge to move and catchiness in 1 s condition predicted by rhythmic information conditions (1.5 or 2 beat stimuli).

DV	*B_1.5 beats_*	SE	95% CrI	BF	*R* ^2^_m_	Structure
Urge to move_1s_	**−0.363**	0.125	−0.608, −0.118	**999**	**0.013**	PS/SI
Catchiness_1s_	0.000	0.099	−0.193, 0.195	1.024	0.000	PI/SI

PI, by-participant intercept; SI, by-stimulus intercept, PS, by-participant slope. Bayes factors reflect evidence for directional hypotheses B < 0.

Bayes factors > 10 are shown in italics, and > 30 in bold.

### Differences in promoting factors

#### Tempo

Regression models revealed that slower tempo reduced urge to move but had no effect on catchiness in the 10-s condition (Table 7). Comparing the numbers in Tables 6, 7 suggests that added rhythmic information in the 1-s stimuli might contribute beyond the general influence of tempo found for the 10-s stimuli. To assess whether this is true, we calculated a model with combined outcomes, which allows to compare whether the effect of tempo was larger on 1-s compared to 10-s urge to move ratings. The evidence that urge to move_1s_ is influenced more by the tempo manipulation is only moderate (BF = 5.359). Hence, we have no clear evidence that the amount of rhythmic information is important beyond tempo.

**Table 7:** Models for urge to move and catchiness in 10s condition predicted by tempo (90 BPM or 120 BPM).

DV	*B_90BPM_*	SE	95% CrI	BF	*R* ^2^_m_	Structure
Urge to move_10s_	**−0.279**	0.135	−0.545, −0.010	**77.431**	**0.007**	PS/SI
Catchiness_10s_	−0.009	0.099	−0.205, 0.184	1.148	0.000	PI/SI

PI, by-participant intercept; SI, by-stimulus intercept; PS, by-participant slope. Bayes factors reflect evidence for directional hypotheses B < 0.

Bayes factors > 10 are shown in italics, and > 30 in bold.

#### Excursion: style bias

Investigating listener-related factors for urge to move and catchiness, which have been shown to overlap (Bechtold et al., 2023, 2024), is not the focus of this study. Hence, the models so far only included a by-participant intercept or slope. However, a participant’s taste or style bias is important to include when modeling the effect of styles (H_IIb_), and potentially for explaining differences in ratings for the short stimuli. Therefore, we calculated these respective models (Table 8). The results show that a participant’s style bias positively influences their ratings. The 1-s results have a limitation: participants indicated the style in response to the 10-s stimuli, and not the 1-s ones. Hence, the assigned styles (and thus by extension the style bias) for the 1-s stimuli is only an extrapolation.

**Table 8:** Models for urge to move and catchiness in 1 s and 10s conditions predicted by style bias.

DV	*B*	SE	95% CrI	BF	*R* ^2^_m_	Structure
Urge to move_1s_	**0.147**	0.024	0.101, 0.193	**> 1,000**	**0.022**	PI/SI
Urge to move_10s_	**0.204**	0.039	0.127, 0.279	**> 1,000**	**0.043**	PS/SI
Catchiness_1s_	**0.195**	0.024	0.147, 0.243	**> 1,000**	**0.039**	PI/SI
Catchiness_10s_	**0.253**	0.037	0.181, 0.324	**> 1,000**	**0.066**	PS/SI

DV, dependent variable, IV, independent variable, PI, by-participant intercept, SI, by-stimulus intercept, PS, by-participant slope. Bayes factors reflect evidence for directional hypotheses B > 0.

Bayes factors > 10 are shown in italics, and > 30 in bold.

#### Musical styles

The participants assigned each 10s stimulus to one or more of 13 musical styles. Table 9 gives the results of the two regression models with these individual assignments as predictors for urge to move and catchiness, and controlled for style bias. As the predictors showed mostly 0 values (indicating that a style has not been chosen for the respective observation), these models required informative priors, and we specified them to expect zeros and small effects.

**Table 9:** Fixed effects of musical styles and style bias on urge to move and catchiness in the 10s condition.

Style	Move				Catchiness			
	*B*	SE	95% CrI	BF	*B*	SE	95% CrI	BF
Country/Western	0.072	0.095	−0.112, 0.260	3.449	−0.035	0.095	−0.220, 0.151	1.779
Downtempo/Ambient	**−0.464**	0.090	−0.644, −0.290	**> 1,000**	**−0.222**	0.093	−0.403, −0.037	**116**
EDM/Dance	0.064	0.092	−0.113, 0.245	3.180	−0.086	0.094	−0.268, 0.101	4.548
Folk/Traditional	0.050	0.102	−0.149, 0.251	2.225	−0.038	0.106	−0.244, 0.166	1.782
Hard Rock/Metal	0.093	0.098	−0.097, 0.286	4.922	**0.293**	0.101	0.094, 0.491	**443**
HipHop/Cont. R&B	−0.008	0.107	−0.216, 0.206	1.129	*0.148*	0.110	−0.064, 0.362	*10.127*
Jazz/Blues	0.024	0.085	−0.147, 0.192	1.588	−0.034	0.088	−0.205, 0.141	1.881
Latin	−0.055	0.155	−0.360, 0.253	1.808	−0.029	0.162	−0.345, 0.282	1.315
Pop/Mainstream	−0.012	0.092	−0.190, 0.168	1.263	**−0.212**	0.093	−0.397, −0.030	**89.909**
Punk/Alternative	0.015	0.141	−0.196, 0.231	1.247	−0.103	0.114	−0.328, 0.120	4.366
Reggae/Dancehall	0.080	0.109	−0.196, 0.355	2.493	0.155	0.150	−0.145, 0.451	5.645
Rock/Rock ‘n’ Roll	−0.003	0.086	−0.170, 0.169	1.057	0.010	0.088	−0.162, 0.183	1.160
Soul/Funk	0.116	0.099	−0.076, 0.308	7.180	*0.145*	0.103	−0.059, 0.350	*11.480*
Style Bias	**0.244**	0.024	0.198, 0.290	**> 1,000**	**0.268**	0.025	0.219, 0.316	**> 1,000**

Standardized regression coefficients, standard errors, and 95% credible intervals are taken from two Bayesian multilevel models with by-participant and by-stimulus random intercepts. Bayes factors reflect evidence for directional hypotheses (B > 0 or B < 0).

Bayes factors > 10 are shown in italics, and > 30 in bold.

Eight styles showed no relevant effect at all: Country/Western, EDM/Dance, Folk/Traditional, Jazz/Blues, Latin, Punk/Alternative, Reggae/Dancehall, Rock/Rock ‘n Roll. Some of these (e.g., Latin and Punk/Alternative) were chosen only rarely. This manifested in large standard errors, which might explain the absence of evidence for these styles. We found negative effects for Downtempo/Ambient for both urge to move and catchiness. Four styles showed an effect on catchiness, while not affecting the urge to move: Pop/Mainstream was negatively associated with catchiness, while Hard Rock/Metal, Soul/Funk, and HipHop/Contemporary R&B showed positive effects or trends. No style affected the urge to move but not catchiness. In summary, five out of 13 assigned musical styles showed effects or trends, and in four cases in a diverging way.

#### Recognizability

Participants’ assessments of how sure they were that an excerpt had appeared before influenced both ratings (Table 10). The estimate and coefficient of determination suggest that recognizability influences catchiness more than urge to move. To assess this, we calculated a model that combines the outcomes, and found extreme evidence that the effect of recognition is stronger for catchiness than for urge to move (BF > 1,000).

**Table 10:** Models for urge to move and catchiness in 10s condition predicted by individual recognizability of the stimulus.

DV	*B*	SE	95% CrI	BF	*R* ^2^_m_	Structure
Urge to move_10s_	**0.049**	0.025	0.001, 0.097	**43.378**	**0.002**	PI/SI
Catchiness_10s_	**0.291**	0.036	0.222, 0.360	**> 1,000**	**0.094**	PS/SI

PI, by-participant intercept; SI, by-stimulus intercept; PS, by-participant slope. Bayes factors reflect evidence for directional hypotheses B > 0.

Bayes factors > 10 are shown in italics, and > 30 in bold.

#### Audio features

We calculated separate regression models for each of the 18 measured audio variables to determine whether each variable influences urge to move, catchiness, or both. This approach allowed us to assess each audio feature effect independently, avoiding confounding due to correlations among predictor variables, and to examine whether the relationship between urge to move and catchiness is constrained by these musical features. The results of the fixed effects from these models are shown in Table 11. However, because the models are separate rather than combined into one, direct comparisons of effect sizes across outcomes are not advised. To evaluate whether a variable affects both outcomes, we checked for the presence of effects in both the urge to move and catchiness models without implying statistical equivalence or difference.

**Table 11:** Models for urge to move and catchiness in 10s condition predicted by 18 audio features.

IV	DV	*B*	SE	95% CrI	BF	*R* ^2^_m_	Structure
HCDF	Urge to move	**0.135**	0.035	0.067, 0.204	**> 1,000**	**0.018**	PS/SI
	Catchiness	**0.099**	0.034	0.030, 0.167	**443**	**0.010**	PS/SI
Entropy	Urge to move	**0.106**	0.040	0.027, 0.185	**210**	**0.011**	PS/SI
	Catchiness	**0.093**	0.037	0.023, 0.168	**185**	**0.009**	PS/SI
Event density	Urge to move	0.036	0.040	−0.041, 0.114	4.583	0.002	PI/SI
	Catchiness	0.018	0.038	−0.058, 0.093	2.152	0.001	PS/SI
Inharmonicity	Urge to move	**0.111**	0.035	0.043, 0.180	**> 1,000**	**0.012**	PI/SI
	Catchiness	*0.057*	0.032	−0.008, 0.121	*24.078*	*0.003*	PI/SI
Key strength	Urge to move	−0.043	0.037	−0.115, 0.030	7.333	0.002	PI/SI
	Catchiness	**−0.061**	0.032	−0.124, 0.003	**30.250**	**0.004**	PI/SI
Novelty	Urge to move	**0.088**	0.036	0.018, 0.159	**126**	**0.008**	PI/SI
	Catchiness	*0.054*	0.033	−0.011, 0.119	*18.093*	*0.003*	PI/SI
Percussiveness	Urge to move	**0.109**	0.035	0.041, 0.176	**666**	**0.012**	PI/SI
	Catchiness	**0.081**	0.031	0.019, 0.143	**172**	**0.007**	PI/SI
Pulse clarity	Urge to move	*0.072*	0.041	−0.007, 0.152	*25.578*	*0.005*	PS/SI
	Catchiness	−0.004	0.036	−0.075, 0.067	1.189	0.001	PS/SI
P. c. entropy	Urge to move	0.002	0.037	−0.071, 0.076	1.094	0.001	PI/SI
	Catchiness	0.020	0.033	−0.044, 0.084	2.705	0.001	PI/SI
RMS loudness	Urge to move	0.028	0.042	−0.055, 0.111	3.053	0.001	PS/SI
	Catchiness	−0.010	0.036	−0.080, 0.062	1.608	0.001	PS/SI
Roll off	Urge to move	**0.123**	0.038	0.049, 0.196	**999**	**0.015**	PS/SI
	Catchiness	**0.080**	0.037	0.008, 0.152	**66.227**	**0.006**	PS/SI
Roughness	Urge to move	*−0.060*	0.036	−0.132, 0.012	*20.220*	*0.004*	PI/SI
	Catchiness	*−0.064*	0.036	−0.134, 0.007	*23.397*	*0.004*	PS/SI
SBF 2	Urge to move	−0.001	0.038	−0.075, 0.076	1.038	0.001	PS/SI
	Catchiness	0.012	0.039	−0.064, 0.089	1.613	0.001	PS/SI
SBF 3	Urge to move	0.011	0.037	−0.062, 0.084	1.593	0.001	PS/SI
	Catchiness	0.012	0.042	−0.070, 0.094	1.550	0.001	PS/SI
SBF 7	Urge to move	0.011	0.038	−0.065, 0.087	1.621	0.001	PS/SI
	Catchiness	0.033	0.044	−0.054, 0.121	3.464	0.002	PS/SI
SBF 9	Urge to move	*0.072*	0.044	−0.016, 0.159	*18.950*	*0.005*	PS/SI
	Catchiness	*0.065*	0.041	−0.018, 0.146	*16.621*	*0.004*	PS/SI
Tonality	Urge to move	**−0.071**	0.036	−0.142, 0.001	**36.915**	**0.005**	PI/SI
	Catchiness	−0.039	0.033	−0.105, 0.025	8.143	0.002	PI/SI
Zero crossing	Urge to move	**0.094**	0.040	0.015, 0.173	**97.765**	**0.009**	PS/SI
	Catchiness	**0.080**	0.037	0.009, 0.156	**66.797**	**0.006**	PS/SI

DV, dependent variable; IV, independent variable; PI, by-participant intercept; SI, by-stimulus intercept, PS, by-participant slope. Bayes factors reflect evidence for directional hypotheses (B > 0 or B < 0).

Bayes factors > 10 are shown in italics, and > 30 in bold.

Six variables showed no effect, conflicting with our expectations: event density, pulse clarity entropy, RMS loudness, SBF 2, SBF 3, SBF 7. Nine variables affected both urge to move and catchiness positively: HCDF (harmonic change detection function, the flux of the tonal centroid), audio signal entropy, amount of high-frequency content (measured by roll off and zero crossing rate), and percussiveness. Spectral inharmonicity and novelty (the probability of consecutive moments) had positive effects on urge to move, and positive trends for catchiness. SBF 9 (sub-band flux no. 9, 6,400–12,800 Hz) showed positive trends, and roughness showed negative trends for both ratings.

In contrast, three variables showed effects on only one rating. Key strength was negatively associated with catchiness but showed no effect on urge to move. Tonality – the difference between the most common and least common pitches – negatively affected urge to move but did not impact catchiness. Lastly, pulse clarity showed a positive trend on urge to move. In summary, 15 variables showed effects (including trends) or null effects on both ratings, while only tonality, key strength, and pulse clarity showed divergent results.

## Discussion

### Conceptual differences

In this study, we investigated a condition under which we expected that experiences of urge to move and catchiness would diverge: very short excerpts of popular music. We expected that an urge to move requires a longer duration, because it is elicited by rhythmic patterns, (violation of) regularity/meter, or repetition (e.g., Witek et al., 2014; Senn et al., 2018). Catchiness, in contrast, is said to act more immediately (Burgoyne et al., 2013; Bechtold et al., 2023). However, our results showed co-occurrences of urge to move and catchiness in very short clips. Hence, our hypothesis H_Ia_ was only partially confirmed: urge to move for short stimuli was lower, but nonetheless present, while catchiness was on average rated similarly to longer stimuli – as expected, and similarly to pleasure. We could confirm our hypothesis H_Ib_: increased rhythmic information led to higher urge to move, while catchiness was unaffected. Together with the weaker correlations in the short compared to the long condition, these duration effects on urge to move but not catchiness revealed conceptual differences.

#### On catchiness

We confirmed that catchiness is an immediate reaction to music. This notion has been reported in Bechtold et al. (2023) and is known from recognition tasks in which listeners identify songs within fractions of a second (Burgoyne et al., 2013; Korsmit et al., 2017; Krumhansl, 2010; Kuiper et al., 2021). What made the short clips catchy? They did not feature a full melody, pattern, or hook, which require more time to unfold, and the music was unfamiliar, preventing explicit recognition. Potentially, an interaction between timbre (i.e., soundscape, instruments), immediately perceived genre (Gjerdingen and Perrott, 2008; Krumhansl, 2010; Mace et al., 2012), and listener taste (i.e., their style bias, which influenced short and long condition ratings) could explain participants’ reactions.

On average, there was no difference between the ratings for the short and long clips for catchiness, indicating that longer music is not necessarily perceived as catchier than the impression formed in the first second. However, the findings are more complex, as the correlation between the ratings for short and long clips was only moderate. This means that the immediate reaction to the first second was often not sustained: music that was initially perceived as catchy often turned out to be not catchy when played for longer and vice versa – participants’ perception changed with more information or duration. This finding aligns with differences in musical structure: often-associated parameters, such as melody, could only unfold and influence participants’ responses in the longer clips, whereas catchiness in the short stimuli depended on other features (see above). Hence, there appear to be two types of catchiness, each relying on distinct aspects of the music: one immediate reaction, and one that needs to be sustained. Similar distinctions exist for hooks and attention. Burns (1987) categorized hooks as textual (e.g., derived from musical structures) or non-textual (e.g., derived from timbre), and studies in other fields have discriminated between transient stimulus-driven attention and goal-oriented sustained attention (Nakayama and Mackeben, 1989; Eimer and Forster, 2003; Smid et al., 2006; Sanchez-Lopez et al., 2014). With these distinctions, we can explain our results: the short stimuli may have relied mostly on non-textual hooks that led to transient attention and non-textual ‘transient catchiness’, while the long stimuli may have relied on textual hooks that elicited sustained attention and textual ‘sustained catchiness’. Because of these differing musical factors, not every pattern promoted strong transient catchiness that was followed by sustained catchiness, which may explain the relatively low correlation between catchiness ratings across duration conditions.

#### On urge to move

Our results regarding urge to move contradict common conception that urge to move is elicited by rhythmic aspects of the music. One second of music cannot convey rhythmic patterns, meter (and thus tempo), or sense of regularity. Yet, although participants on average experienced less urge to move compared to the longer clips, their ratings remained well above zero. When the short clips encompassed 2 beats instead of 1.5, the gap in ratings to the longer clips narrowed, suggesting that the threshold for experiencing an urge to move not constrained by duration may only be slightly longer than the 2 beats we used in this study: a full bar (2–3.33 s) may have been sufficient to close the distance to the 10-s ratings. The two duration conditions were strongly correlated, suggesting that the urge to move in the first second was indicative of how listeners react to longer clips. Interestingly, while pleasure and urge to move are often closely related in groove research or form the two components of the concept, we found a difference here, as pleasure was similarly high on average for both durations.

There is a potential methodological limitation to these results. We measured urge to move with a self-report questionnaire: participants first heard the music and afterwards answered questions, i.e., the ratings are conscious judgements made after the experience. It is possible that participants’ answers captured a desire to move if the music were to continue, instead of the actual felt urge to move while listening for 1 s. In other words, the one-second clip resembled music that, if it were to continue, would likely elicit an urge to move. Participants were instructed both in the introduction and the prompts to each rating to rate their actual response to the clip, but it cannot be ruled out that some participants may have responded in this more analytical way. Furthermore, if we consider our measure of urge to move in the context of the more holistic groove experience, which also includes participation and immersion (Duman et al., 2024; Bechtold et al., 2023) or state of being (Danielsen, 2006), one second is hardly enough to elicit these more temporally distributed responses. Hence, in this study, the measured urge to move is representative only for the urge to move aspect of groove, and not groove more widely.

With that said, what structural elements in these short clips made participants want to move? Due to the short duration, it is unlikely that features such as syncopation (e.g., Witek et al., 2014), microtiming (Frühauf et al., 2013), nuances (Roholt, 2014), pattern types (Senn et al., 2018; Sioros et al., 2022), or violation of expectations (e.g., Stupacher et al., 2022b) played a role. Increased rhythmic information (2 beats vs. 1.5 beats)—and the resulting increased chance to grasp a regularity, known to foster an urge to move (Senn et al., 2023a, 2024; Jerjen et al., 2024)—constituted an effect of musical structure. Relatedly, the experienced urge to move could have been a result of trying to find the beat through movement or from motor cortex activation (Grahn and Rowe, 2013). Aside from that, the urge to move could have been elicited by instantly perceived factors, such as timbre, genre (Gjerdingen and Perrott, 2008; Krumhansl, 2010; Mace et al., 2012), affect (Peretz et al., 1998), emotions (Krumhansl, 2010), and their potential interaction with listeners’ taste and background. However, musical styles barely influenced the urge to move in this study (see below). Additionally, energetic arousal could have been involved, which acts fast and has been shown to promote an urge to move (Senn et al., 2023a, 2024; Bechtold et al., in press; Jerjen et al., 2024). This could also explain the correlation we found between the ratings for short and long clips: the induced energy could have been similar, regardless of stimulus duration. Another potential reason, which aligns more closely with this study’s focus as it addresses the relationship between catchiness and urge to move, is transient catchiness (see below).

#### On the relationship between urge to move and catchiness

We found that co-occurrence between urge to move and catchiness was rarer in the very short stimuli (*r* = 0.402) compared to the longer ones in the present (*r* = 0.487) and other studies (Bechtold et al., 2024: *r* = 0.657; Bechtold et al., 2025a: *r* = 0.475–0.600). This demonstrates the diverging sensitivity to duration and thus conceptual differences between urge to move and catchiness. The respective correlations between long and short clips for the two experiences revealed a further difference: participants often revised their initial impression of catchiness when the music continued, which we explained with two related but different aspects of catchiness, whereas the first impression ratings for urge to move, while generally lower, were more stable if the music continued.

The present co-occurrences even in short stimuli suggest a causal relationship. We found that the first second’s (= transient) catchiness can explain around 10% of the variance in the first second’s urge to move ratings. Based on Bechtold et al. (2024), who found that catchiness influences urge to move via pleasure in longer clips, we performed a mediation analysis which confirmed that this pathway also applies to the responses to the 1-s stimuli. This pathway helps explain the non-zero ratings in response to short clips, at least partially. Since the first second is part of the longer clips, this also affects the respective longer ratings. Here, transient catchiness explains around 8% of the variance in the longer urge to move ratings. Conversely, the experienced urge to move to the longer clips could explain 20% of the sustained catchiness. Consequently, we can speculate about a reinforcing loop: transient catchiness fosters an urge to move via pleasure, and when this urge to move is sustained (e.g., through musical structure or performance), it fosters sustained catchiness. This back-and-forth – or, less speculatively, the found co-occurrence – limited our ability to clearly identify the boundaries of the relationship between the urge to move and catchiness. It should be noted, however, that the observed relationship between urge to move and pleasure might be slightly inflated due to proximity bias, as both ratings were given on the same page, whereas catchiness was always rated separately. Future research aiming to explore the boundaries of the relationship between groove and catchiness may require a different context in which the two are presumably less related than in popular music, e.g., music not primarily designed for dancing or memorability.

### Differences in promoting factors

We investigated four different types of music-related factors to identify differences in how they influence urge to move and catchiness. We first discuss the clearer findings (tempo, recognizability), followed by the more complex ones (musical styles, audio features).

#### Tempo

As expected, the 120 BPM stimuli elicited a stronger urge to move for the 10-s stimuli, while catchiness was not affected by tempo (H_IIa_). The results for urge to move corroborate Etani et al.’s (2018) optimal tempo range and Jerjen et al.’s (2024) finding that faster tempos increase the experience of groove. While the effect was small, this supports the conclusion that, despite the relationship between catchiness and urge to move, different aspects of the music are responsible for promoting these experiences.

#### Recognizability

Recognizability showed a positive effect on both urge to move and catchiness, aligning with Bechtold et al. (2024), who also found positive effects of recognition on catchiness, pleasure, and urge to move. However, the effect was minimal on urge to move and substantial for catchiness. This was not unexpected, given the conceptual overlap between recognition and catchiness, and confirms H_IIc_. In consequence, recognizability of music can be considered a divergent factor: it is more important for catchiness than for the urge to move.

#### Musical styles

In our study, participants assigned each 10-s stimulus to one or more of 13 popular music styles. We analyzed how the individual style assignments affected urge to move and catchiness with participants’ style bias controlled. For most styles, evidence was very low, but five styles affected the ratings. Four of these affected only catchiness but not the urge to move. Thus, the perceived style of music promoted urge to move and catchiness differently in some cases, partially confirming H_IIb_. Downtempo/Ambient was the only style to affect urge to move, reducing it, and it also reduced catchiness. This is straightforward since Downtempo/Ambient music is not geared towards dancing or sticking in mind. Contrary to our expectations, other styles did not affect urge to move, including the frequently studied funk (e.g., Danielsen, 2006; Senn et al., 2021; Stupacher et al., 2023), EDM/Dance (e.g., Wesolowski and Hofmann, 2016; Lustig and Tan, 2019; Duncan and Orgs, 2024), and Pop/Mainstream, which contradicts previous research looking at broader style families (Senn et al., 2021; Stupacher et al., 2023). Pop/Mainstream showed a negative association with catchiness, despite its assumed importance for this style (Rösing, 1996; Bechtold et al., 2023). It may be that the AI-generated Mainstream stimuli sounded too conventional to be catchy. We also found positive effects of Hard Rock/Metal, and positive trends of Soul/Funk and HipHop/Contemporary R&B on catchiness. Potentially, these stood out more from the other stimuli and were thus perceived as more memorable and distinctive. More generally, participants might have had difficulties classifying the music or envisioned different music when indicating their style preferences compared to our stimuli. In sum, these findings showed that perceived musical style can be a factor for why music promotes catchiness without promoting an urge to move, even when controlling for the listener’s taste. However, no style exhibited opposing effects on urge to move and catchiness.

#### Audio features

We measured 18 audio features in the 10-s clips and examined their effects on urge to move and catchiness. Nine influenced both responses, three influenced only one, and six had no effects. Hence, we could only partially confirm H_IId_: some musical characteristics promoted either urge to move or catchiness but not the other, while many affected both or neither.

Among the three with diverging effects, only pulse clarity behaved as expected in that a clearer pulse facilitated an urge to move (as in Madison et al., 2011; Stupacher et al., 2016) while not affecting catchiness. For the pitch-related key strength and tonality, we expected positive effects on catchiness and none on urge to move. However, lower key strength, i.e., a less affirmed key, increased catchiness, possibly because of the frequent use of some out-of-key notes (e.g., flat 7) or chords in popular music (Moore, 1995; Temperley and de Clercq, 2013). Stronger tonality, i.e., more diatonic music, unexpectedly reduced urge to move. The positive effects of HCDF, entropy, or novelty on both response variables also relate at least partially to pitch. These findings suggest that pitch-related features, typically overlooked in groove research, may play a role in movement-related responses. We also corroborated some of the previous effects on urge to move (e.g., for inharmonicity, percussiveness, or zero crossing rate), and showed that these also affect catchiness.

Despite being selected based on prior findings, several variables showed no effect in this study. This might have been due to the stimuli: in contrast to studies that controlled specific variables or manipulated music in a specific way (Burger et al., 2013; Wesolowski and Hofmann, 2016; Lustig and Tan, 2019; Duncan and Orgs, 2024; Bechtold et al., in press), our stimuli varied in musical styles and many other parameters. Yet, some results (positive effects of SBF 2, event density, and RMS loudness on urge to move) have been found for non-manipulated music (Stupacher et al., 2016; Bechtold et al., 2025b), which makes the respective null effects in our study puzzling.

Taken together, the results of the objective audio features are complex. We identified variables that affected catchiness, and corroborated some results on urge to move, but also found unexpected outcomes for the latter – either pitch-related effects or null effects. These results require further clarification, especially across different contexts: the influence of pitch on urge to move warrants more investigation, and the catchiness findings need replication, given differences in measurement and definitions (Van Balen, 2016; Kuiper et al., 2021; Silas and Müllensiefen, 2023). For this study’s aim, exploring the limits of the relationship between urge to move and catchiness, the audio features were partially expedient. While some musical characteristics promote one but not the other, many influenced both or neither. This may stem in part from the causal link between urge to move and catchiness in the 10-s stimuli: if one leads to the other, there is limited room for finding distinct musical causes. This space may have been occupied by more consciously perceived characteristics, such as tempo or rhythmic information, compared to, for example, perceived fullness (e.g., fluctuation in bass frequencies) or brightness (e.g., the music’s noisiness).

## Conclusion

In this study, we examined the relationship between urge to move and catchiness by focusing on their differences and exploring a context in which they were presumably unrelated. We investigated extremely short durations to reveal conceptual differences, and analyzed a variety of music-related factors to determine which characteristics makes music likely to promote one but not the other.

We found that catchiness is an immediate reaction to music but is surprisingly prone to revision when the music continues, which we attributed to two distinct aspects: transient and sustained catchiness. As expected, urge to move was lower in response to very short and minimally rhythmically informative stimuli. Yet, listeners nonetheless experienced some urge to move, which we suggest might be partially explained by the transient catchiness that these clips induced. This illustrates a dilemma of this study: while we aimed to uncover differences and a condition in which the two diverge, we still found them correlated, and ended up with a causality between urge to move and catchiness as likely explanation. As such, the study was only partially successful in identifying the limits of their relationship. Future research may be more successful in disentangling them by using a different musical context than popular music or other measurement methods. The distinction between transient and sustained catchiness warrants further study, as does their respective influence on the urge to move. Moreover, the duration thresholds for experiencing an urge to move should be determined.

We were able to show that the relationship between the urge to move and perceived catchiness is constrained by musical characteristics, as we identified musical features that promote one but not the other. However, we found no directly opposing effect, i.e., no feature that promoted one while simultaneously hindering the other. Tempo influenced the urge to move but not catchiness, while recognizability affected catchiness much more than the urge to move. Other results were more complex: some popular music styles were associated with catchiness but not the urge to move, while many styles showed no effect on either. Similarly, most audio features showed effects – or not – on both outcomes, and only a few can be used to distinguish between groovy and catchy music. These audio features require further study: the role of pitch for urge to move remains unexplored, and findings on catchiness require replication. In summary, our results show that musical factors play a role in shaping whether listeners experience music as catchy or groovy, both, or neither. This complements previous results, which showed that listener-related factors—such as familiarity, taste, or expertise—tend to promote either both or none, but not one without the other. In other words, a key difference between urge to move and catchiness lies in the music that promotes them.

This study indicates that future research that examines why catchiness or an urge to move in response to music come about should account for the potential influence of the other, as the two appear to be causally related, even under extreme conditions.

## Data Availability

The data, analyses, and materials presented in this study can be found in an online repository at: https://osf.io/42yd6/.

## Footnotes

^1^ https://osf.io/42yd6/

^2^ www.soscisurvey.de
